# Sleep Disturbances in Patients with Persistent Delusions: Prevalence, Clinical Associations, and Therapeutic Strategies

**DOI:** 10.3390/clockssleep2040030

**Published:** 2020-10-16

**Authors:** Alexandre González-Rodríguez, Javier Labad, Mary V. Seeman

**Affiliations:** 1 Department of Mental Health, Parc Tauli University Hospital, Autonomous University of Barcelona (UAB), I3PT, Sabadell, 08280 Barcelona, Spain; agonzalezro@tauli.cat; 2 Department of Psychiatry, Hospital of Mataró, Consorci Sanitari del Maresme, Institut d’Investigació i Innovació Parc Tauli (I3PT), CIBERSAM, Mataró, 08304 Barcelona, Spain; labadj@gmail.com; 3 Department of Psychiatry, University of Toronto, #605 260 Heath St. West, Toronto, ON M5T 1R8, Canada

**Keywords:** insomnia, sleep disorders, delusions, delusional disorder, psychosis

## Abstract

Sleep disturbances accompany almost all mental illnesses, either because sound sleep and mental well-being share similar requisites, or because mental problems lead to sleep problems, or vice versa. The aim of this narrative review was to examine sleep in patients with delusions, particularly in those diagnosed with delusional disorder. We did this in sequence, first for psychiatric illness in general, then for psychotic illnesses where delusions are prevalent symptoms, and then for delusional disorder. The review also looked at the effect on sleep parameters of individual symptoms commonly seen in delusional disorder (paranoia, cognitive distortions, suicidal thoughts) and searched the evidence base for indications of antipsychotic drug effects on sleep. It subsequently evaluated the influence of sleep therapies on psychotic symptoms, particularly delusions. The review’s findings are clinically important. Delusional symptoms and sleep quality influence one another reciprocally. Effective treatment of sleep problems is of potential benefit to patients with persistent delusions, but may be difficult to implement in the absence of an established therapeutic relationship and an appropriate pharmacologic regimen. As one symptom can aggravate another, comorbidities in patients with serious mental illness all need to be treated, a task that requires close liaison among medical specialties.

## 1. Introduction

Delusions are core symptoms of all psychotic illnesses, and they specifically characterize one of the less well-known forms of psychosis, delusional disorder (DD). This condition usually presents with fixed monosymptomatic delusions; thought disorders, hallucinations, mood disturbances and negative symptoms may occur, but are generally absent [1]. (Table 1). The current Diagnostic and Statistical Manual of Mental Disorder, Fifth Edition (DSM-5) describes seven subtypes of DD, grouped according to the content of the primary delusion [1]. (Table 2).

The overlap of DD with schizophrenia is considerable, which is why the nosological status of DD continues to be the subject of much debate [2]. While it is currently considered a diagnostic entity of its own [3], DD has been seen by many classical writers not only as closely related to schizophrenia, but also to affective disorders, especially mania [4]. However, the vast majority of the scientific literature today supports the nosological validity of DD, highlighting demographic and clinical differences vis à vis other psychotic disorders, for instance its characteristic emergence in middle to late life and the relative preservation of good social functioning throughout the course of illness [5].

While sleep disturbance in the context of DD has not been systematically studied, almost all psychiatric syndromes, including those where delusions are prominent, are associated, to a degree, with difficulties in sleep. This is either because risk factors for poor sleep and poor mental health are similar or because symptoms of mental illness, such as delusions, lead to sleep disturbance, or because disturbed sleep instigates psychopathology, such as the development of delusions. The close connection between symptom severity and sleep quality has been demonstrated in many psychiatric syndromes.

### 1.1. Aims

The aim of this review was to explore the connection between chronic delusions and sleep, with a particular focus on the nosological category of DD. Limiting the search to DD is impossible because the extant literature on sleep and DD contains only case descriptions, no case series and no clinical trials. As a result of of this, we first probed the sleep-delusion connection in psychotic disorders, such as affective disorders with psychotic features and schizophrenia, since both these conditions are characterized by delusions. We also tried to determine whether the emergence or severity of individual symptoms often seen in psychotic illnesses (delusions, but also cognitive distortions, emotional distress, misperceptions, suicidal ideation) affect sleep or are affected by problems of sleep. In addition, our aim was to examine the potential role of therapeutic medications in the induction of sleep problems such as insomnia, hypersomnia, sleep apnea, sleepwalking, restless legs syndrome, and bruxism. Finally, our aim was to evaluate the influence of established sleep therapies, in particular those that have been developed for insomnia and nightmares, on delusions and related psychotic symptoms.

### 1.2. Method

This is not an exhaustive or systematic review. Due to the fact that the topic of delusional disorders has received little specific attention in the sleep research area, this narrative-style look at the literature on sleep in related psychotic illness attempts to draw parallels that might help to advance the field. It is based on electronic searches, conducted in PubMed and Google Scholar databases, of recent work published in English, French, and Spanish, using the following search terms: Delusional Disorder AND Sleep; Psychosis AND Sleep; Mental symptoms AND Sleep; Affective Disorder AND Sleep; Schizophrenia AND Sleep; Antipsychotic medication AND Sleep; Sleep disturbances AND Treatment. We initially looked only at publications of the last two years and then searched the titles in the reference sections of retrieved articles to obtain older publications especially pertinent to our aims. For the final selection, we chose the most recent articles that contained relevant up-to-date information on our preselected subtopics.

This review has been divided into the following sections: (a) sleep and mental disorders, (b) sleep and psychosis (bipolar disorder, schizophrenia and DD), (c) sleep and psychotic symptoms, (d) medications and sleep problems, and (e) therapeutic options for sleep disorders in patients with fixed delusions.

## 2. Sleep and Mental Disorders

Evidence for associations between sleep quality, sleep disturbances and prevalence, symptom severity or treatment response in mental disorders has been poorly integrated, and the neurobiological basis of the sleep/mental illness connection remains unknown. It is, however, well accepted that sleep disorders are commonly comorbid with mood disorders. Sleep problems are considered independent risk factors for the occurrence of depressive episodes and for poor response of depression to both pharmacological and nonpharmacological treatments [6]. Sleep loss or insomnia has received particular attention in the mental health–sleep field and several helpful guidelines have been developed for the assessment and treatment of insomnia [7].

Schennach and collaborators [6] carried out a naturalistic study of 5481 psychiatric inpatients in order to explore the association of sleep quality, sleep disturbances, and psychiatric treatment outcomes in the hospital setting. Sleep was assessed by means of the Pittsburg Sleep Quality Index (PSQI) questionnaire and depressive symptoms were evaluated by the Beck Depression Inventory (BDI) at admission and at study endpoint. Sleep disturbances were very prevalent. The main result was that PSQI scores improved from admission to hospital discharge, perhaps because psychiatric symptoms improved, or because regular hospital routines entrained circadian rhythms, all of which suggests that sleep disturbances were secondary phenomena. Patients with obsessive–compulsive disorder (OCD) showed the least sleep disturbances overall, suggesting that their underlying condition was not as closely linked to sleep as are other psychiatric illnesses. Patients with post-traumatic stress disorder (PTSD) showed the same degree of sleep disturbance at baseline as at study completion, suggesting that sleep issues play a more foundational role in this category of illness than in others. While sleep scores improved, the investigators concluded that as many as half of all psychiatric inpatients, even while receiving hospital care, do not attain full remission of their sleep disturbances [6].

A study by Mondal and collaborators [8] examined the prevalence of sleep disorders and the severity of insomnia in psychiatric outpatients. Sleep quality was assessed by means of the PSQI questionnaire and severity of insomnia through the use of the Insomnia Severity Index (ISI) scale. Approximately 83% of this outpatient sample showed at least one type of sleep disorder, 78% presenting symptoms of insomnia. In 29% of these, the insomnia was considered moderate to severe. The other presenting sleep problems were: sleep-related breathing disorders, excessive sleepiness, and disturbed sleep/wake cycles. Most patients fulfilled criteria for at least two parasomnias. Patients diagnosed with a psychotic disorder, for the most part, suffered from subthreshold insomnia, while, in those with a major depressive disorder, insomnia was moderate to severe, confirming the well-known clinical link between insomnia and depression [8].

More recently, Freeman and co-workers [9] reviewed the topic of sleep disturbances and psychiatric disorders to determine whether the relationship was bidirectional. They found that, because sleep disturbances accompany most mental illnesses, their presence/absence cannot be said to specifically characterize any one illness. Furthermore, the review concluded that sleep disturbance should not only be viewed as the secondary effect of a primary illness, that it may instead, according to the evidence, serve as a contributing factor for the emergence of the illness or, at the very least, as an early warning sign. The authors cited recent work that demonstrated the overlap between genetic, neurobiological (circadian rhythm disruptions), and environmental (trauma experiences) factors triggering both sleep difficulties and mental health disorders [9].

## 3. Sleep and Psychosis

A recent editorial reported that the prevalence rate of insomnia, defined here as either nonrestorative sleep or difficulty initiating or maintaining sleep, ranges from 30% to 80% in psychotic illness [10]. Classical theory has considered insomnia to be a consequence of psychotic symptoms rather than a risk factor for their development, but modern psychiatry is beginning to view the relationship as bidirectional [11]. The most recent systematic review of this field [12] identified 37 clinical and 21 nonclinical studies which, together, resulted in the conclusion that sleep quality predicts the onset and also the persistence of psychotic symptoms (e.g., paranoid beliefs (e.g., delusions) and hallucinations) and that negative affect mediates the effect. Sleep disturbance, such as insomnia or nightmares, might, it is now thought, serve as an early warning sign of psychotic relapse or as an indication that ongoing medication needs reconsideration [11]. The view has also been promoted that effective treatment of insomnia or nightmares might be capable of reducing the severity of psychotic symptoms [12].

An observational study of 29 patients with nonaffective psychosis addressed the links among insomnia, negative effect, and psychotic experiences [13]. The hypothesized mediation model that was used showed that insomnia was a strong predictor of hallucinations, but that this was not the case vice versa. A bidirectional relationship was, however, shown between insomnia and paranoid symptoms. This reinforces the hope that treating insomnia could ameliorate specific symptoms of psychosis [14]. This is not a new idea. A sleep cure was the prime treatment of psychosis in Russian psychiatry in the first half of the 20th century [15].

More recently, Kammerer et al., in a cross-sectional survey [16], found that the frequency and degree of distress caused by nightmares was significantly associated, among young adults in the general population, with paranoid thoughts, hallucinations, and negative psychotic symptoms. Kasanova and collaborators [17] investigated the effect of variation of sleep quality specifically on paranoid symptoms in acutely ill individuals with psychotic disorders. They found that poor sleep quality predicted increased severity of paranoid symptoms but only in the morning, suggesting that the effect might be transient.

To sum up this section, a paradox exists between the association of sleep disturbance and psychosis. While the link is generally acknowledged [18], the use of structured sleep assessments and targeted sleep treatments remains relatively rare in this population.

### 3.1. Sleep Disturbances in Bipolar Disorder

Studies of sleep and bipolar disorder have not distinguished between individuals who did and did not, at the time of study, exhibit psychotic features. It is known that, on average, over half of bipolar disorder patients experience mood episodes with psychotic symptoms at least once over a life time (DSM-5) [1]. Whether in conjunction or not with psychosis, most (69%−99%) individuals with bipolar disorder report problems with sleep, the severity of which varies over different phases of the disease [19]. Reduced sleep is a common manifestation of both manic and depressed episodes and sleep disturbances have been associated with remissions and exacerbations of symptoms, with recurrences, and with fluctuations of both social functioning and quality of life. This was confirmed in a recent cross-sectional, naturalistic, multicenter study investigating the link between sleep quality/satisfaction, sleep duration, social function, and quality of life in a sample of 119 Spanish euthymic outpatients with a diagnosis of bipolar disorder [20]. Half of the patients in this study reported at least one sleep complaint, nighttime wakenings and difficulty falling asleep being the most common (60.5% and 31.9%, respectively). The use of benzodiazepines for sleep was negatively correlated with sleep satisfaction and quality of life (QoL) [20]. This was in contrast to the use of anticonvulsants and antipsychotics, which left QoL scores intact.

Investigators have found a correlation between sleep disturbances and psychosocial functioning in patients with bipolar disorder, even during their inter-episodic periods. The aim of a study by Samalin and colleagues [21] of 468 euthymic outpatients with bipolar disorder was to explore the potential correlation among residual or subthreshold depressive symptoms, sleep disturbances, self-perceived cognitive performance, and social functioning. Sleep disturbances were found to be indirectly associated with poor functioning via residual depressive symptoms and subjective perception of cognitive performance. Grover and colleagues [22] conducted a cross-sectional study of 844 individuals diagnosed with bipolar disorder in clinical remission. Residual symptoms following depression were mainly anxiety and impaired insight. Residual symptoms following mania were poor insight and, in almost 25% of cases, sleep disturbance. Inter-episode (psychosis-free, it is reasonable to assume) correlations suggest that, in this condition, sleep disturbances are not directly linked to psychotic symptoms.

A recent review explored the literature on sleep electroencephalography (EEG) in bipolar disorder patients in order to better understand sleep architecture and oscillatory activity across all phases of the illness [23]. Twenty-two studies were included in the review, six inpatients during manic episodes, seven during depressive episodes, seven in euthymic patients, and two in individuals at high risk for bipolar disorder. Total sleep time and sleep latency were found to be impaired in all stages of bipolar disorder. Moreover, increased REM density appeared to precede the onset of the disease. A difficulty initiating sleep was found across all phases of illness [23]. As in other studies, comparisons between patients with and without psychotic symptoms were not made. As a result of this, it is not clear how studies of sleep in bipolar disorder can be extrapolated to delusional disorder.

### 3.2. Sleep Disturbances in Schizophrenia

There is a long and extensive history of literature on sleep disturbances in patients with schizophrenia during actively psychotic states, during at-risk states, and during remission [24,25,26,27,28,29,30,31,32,33,34]. Sleep problems have now begun to be seriously considered as targets for treatment in this condition [35]. Findings from schizophrenia are very relevant to delusional disorders because of the considerable overlap between the two disorders. In schizophrenia, sleep quality has been correlated with the degree of paranoid ideation [36], with hostility and violence [37], with poor quality of life [38,39], with memory functions [40], with physical activity [41], and with dopamine signaling [42]. These correlations may well also apply to DD. In the following subsections, we detail the most frequent sleep disturbances that have been reported in schizophrenia.

#### 3.2.1. Insomnia

One study of first episode psychosis patients (*n* = 280) found that 22.6% suffered from clinical insomnia, especially prominent in individuals who smoked, used alcohol, and were not on antipsychotic treatment [43]. A very similar prevalence (23.2%) has been reported among medicated persons with schizophrenia [38]. Reeve et al. [44], on the other hand, found that the rate of insomnia reached 50% in 60 patients with early psychosis (mostly schizophrenia). In this carefully conducted study, the dose of antipsychotics (APs) made no difference to the insomnia rate despite the fact that many APs are known for their marked sedative properties.

It is difficult to be precise about prevalence rates because insomnia is differently defined and evaluated in different studies and is affected by many factors—age, ambient light and noise level, extent of physical discomfort, use of stimulants, concomitant depression or anxiety [45], and features specific to psychosis, such as severity of intrusive thoughts, delusions, and hallucinations.

#### 3.2.2. Hypersomnia

Nighttime insomnia may, in part, be secondary to sleep during the day due to the effects of antipsychotic medication or the high rate of sleep apnea in schizophrenia. Reeve et al. [44], in the study of early psychosis patients referred to earlier, found a prevalence rate of 23.3% of excessive daytime sleepiness in patients receiving antipsychotic medications. Excessive daytime sedation may also be due to substance abuse and perhaps also to social isolation, loneliness, and a monotonous day routine secondary to the negative symptoms in schizophrenia (apathy, lack of interest in socialization) [46]. Daytime sleepiness may result from disturbed circadian rhythms in some patients with schizophrenia [25,31]. In a very interesting and methodologically excellent study by Wulff et al. [47], the variability of sleep and wake time was found to be significantly greater in a schizophrenia group (*n* = 20) than in a comparable group of unemployed persons (*n* = 21). Intra-individual difference in sleep parameters in the schizophrenia group was two- to threefold greater than that in the control group, individuals with schizophrenia showing inconsistent sleep patterns but, in general, taking longer to fall asleep, spending a longer time in bed, and, in total, sleeping longer than the controls.

#### 3.2.3. Sleep Paralysis

Sleep paralysis is defined as a recurrent inability to move at the beginning of sleep or immediately upon waking. Episodes of immobility are frequently associated with a variety of characteristic hallucinations, such as a sense of an evil presence (known as intruder hallucinations), pressure on the chest (called incubus hallucinations), and illusions of movement (called vestibular–motor hallucinations) [48,49,50].

The presence of sleep paralysis is often overlooked in schizophrenia because the hallucinatory component is seen as an integral part of schizophrenia itself rather than as a comorbidity. This is an illustration of ‘diagnostic overshadowing’ [51], whereby clinicians do not address significant medical comorbidities in persons with serious mental illness, considering them part and parcel of the original disorder.

Among Reeve et al.’s [44] careful study of 60 patients with early psychosis, 15% reported sleep paralysis. Of 71 schizophrenia patients in a study from India, the prevalence of sleep paralysis was also 15% [52], almost twice as high as in the general population (estimated to be 8%) [53].

#### 3.2.4. Sleepwalking and Sleep Terrors

Reeve et al. [44] put the rate of sleepwalking in schizophrenia at 5%. This represents the base rate plus the added risk of sleepwalking induced by various medications [54], among which antipsychotics are major culprits [55]. Drugs promote sleepwalking by interfering with NREM deep sleep. Antipsychotics also induce weight gain and obesity, which is a further risk factor for sleepwalking [56]. Upon waking, sleepwalkers have no memory of what they did during the night so that, when they find that household items have been displaced, they suspect intruders. When persons with schizophrenia voice such suspicions to their psychiatrists, their antipsychotic dose is often increased on the assumption that paranoid symptoms are worsening, which intensifies the problem [57].

Sleep terrors (Reeve et al. [44] set their prevalence at 8.3% in the early psychosis population) can also occur during sleepwalking. This is important clinically as this sleep disturbance can lead to violence upon awakening, with ensuing legal consequences.

#### 3.2.5. Other Parasomnias

Antipsychotics also increase the prevalence of other sleep difficulties in early schizophrenia—sleep apnea (15%), bruxism (18.3%), and restless legs syndrome (23.3%) [44]. These will be further discussed in a later section.

#### 3.2.6. Nightmares

Nightmare disorder is defined as recurrent episodes of intensely disturbing dreams involving negative emotions such as fear, angst, fury, or despair. Reeve et al. [44] found the prevalence in early schizophrenia to be very high (48.3%), with no association to antipsychotic medication. It has been suggested that nightmares in schizophrenia are a continuation into sleep of frightening daytime delusions [58]. This raises the hope that, if nightmares were cured, daytime delusions would also abate [59].

To sum up this section, a number of sleep problems have been found when studying patients with schizophrenia. The findings in schizophrenia may apply to DD, but this speculation needs to be confirmed by more specific evidence.

### 3.3. Sleep Disturbances in Delusional Disorders

To date, there are no research studies on the prevalence of sleep disturbances in DD, or on their impact on the clinical course of the illness [60]. Only case reports on links between sleep parameters and DD are available.

Basu and collaborators [61] reported a case of a 38-year old-woman with DD (subtype: delusional jealousy) who developed restless leg syndrome (RLS) when treated with the AP, olanzapine. She complained of sleep difficulties due to an itching sensation in both upper and lower limbs that occurred only at night. She reported no breathing problems, snoring, or abnormal limb movements. Her International Restless Legs Scale score was 24, which decreased to 12 after switching from olanzapine 15 mg/day to a more potent dopamine blocker, risperidone, which was gradually increased to 6 mg/day as the olanzapine was gradually withdrawn. Olanzapine-induced akathisia was initially considered in the differential, but involvement of the upper limbs, lack of restlessness symptoms during the day, a zero score on the Barnes Akathisia Rating Scale, and improvement on risperidone ruled out akathisia. The RLS appears to have been an idiosyncratic reaction to olanzapine [61].

Khazaie and colleagues [62] reported the case of a 60-year-old woman who was so distressed by her inability to sleep that she attempted suicide. Actigraphy showed normal sleep patterns, which led to a diagnosis of sleep state misperception (purely subjective insomnia). The patient was tried on various medications for depression and given a course of six electroconvulsive treatments. Despite treatment, she continued to complain that she was not sleeping. In the face of this persistence of symptoms, the patient was diagnosed with DD, somatic subtype. She was placed on olanzapine 5 mg/day and gradually improved. She no longer complained of insomnia, either because her psychotic belief responded to olanzapine or because olanzapine proved to be an effective hypnotic. This case illustrates how difficult it is to distinguish a delusion from a misperception of a physiological state [62].

Bottlender and Möller [63] briefly reported a case of DD (jealousy subtype) in a 60-year-old patient whose illness had begun in the previous year and who was being treated with high doses of a combination of antipsychotics, mainly oral and depot haloperidol. The patient showed blunted affect, affective rigidity, restrictive thinking, and social withdrawal, which were initially attributed to her treatment. This was gradually changed to monotherapy with risperidone, 6 mg/day. Delusional symptoms improved, but emotional blunting and related symptoms persisted and, after positive polysomnography, were reattributed to sleep apnea. Positive airway pressure therapy was initiated, with an improvement in the affective blunting and social withdrawal. This case illustrates the ability of treatment for a sleep disturbance to reduce the severity of psychological symptoms. Negative symptoms, such as the ones seen in this case, are considered rare in DD, but they do occur [64].

Sporadic case reports make theoretical concepts memorable, but their lessons cannot easily be generalized.

## 4. Association of Sleep Disturbances with Common Symptoms of Delusional Disorder

While there are no controlled trials of specific sleep impairments in DD patients [65], there are studies that show correlations between sleep problems and individual symptoms. It now seems possible, as has been suggested above in the context of related psychotic illnesses, that sleep disturbances in DD are not necessarily a result of illness—they can antedate illness, increase vulnerability to psychosis, and trigger symptoms.

In brief, sleep quantity and quality has been found to correlate with the severity not only of paranoid ideation, but also of psychotic symptoms in general, subjectively experienced deterioration of cognitive performance, and suicidal thoughts [13,66,67].

### 4.1. Association of Insomnia with Psychotic Symptoms

Recently, Goines and collaborators [66] investigated the effect of sleep disturbances on symptoms in 740 young individuals at clinical high risk for psychosis. Sleep problems were assessed using the general symptoms section of the Scale of Prodromal Symptoms (SOPS) and graded: (0) absent, (1) questionably present, (2) mild, (3) moderate, (4) moderately severe (disruption of several aspects of functioning), (5) severe, and (6) extreme (48 h without sleep). Results showed that sleep problems were positively and significantly associated with symptoms of potential psychosis, especially suspiciousness and perceptual distortions. The more severe the sleep problems, the more severe the prepsychotic symptoms, their relationship mediated by symptoms of depression. This suggests that effective clinical management of sleep disturbances and of depression could help to improve paranoid symptoms and distortions of perception in those at high risk for developing a psychotic illness [64].

In line with these findings, Reeve et al. [13] conducted a randomized controlled trial with 68 nonclinical volunteers to test the hypothesis that insomnia could cause symptoms such as paranoia and hallucinations. In the sleep loss study condition, the average sleep duration was approximately 5 h for two consecutive nights, while in the control condition it was 7 h. Adherence to the protocol was monitored using actigraphic recording, text messages response, and sleep diaries. Psychopathological assessment instruments included the Specific Psychotic Experiences Questionnaire (SPEQ) that measures paranoia, hallucinations, cognitive disorganization, grandiosity, and anhedonia. Affect was assessed by means of the Dunn Worry Questionnaire, perception by the Ebbinghaus Illusion and Perceptual Vigilance Tasks, and decision-making by the Jumping to Conclusions task. Participants in the sleep loss condition reported significant increases in negative affect, which led to paranoia, hallucinations, cognitive disorganization, anxiety, and memory impairment [13].

Similar findings were reported in a community sample of 61 adolescents from Germany. Hennig and Lincoln [67] assessed subjective and objective measures of sleep quality to determine whether low quality sleep led to an increase in paranoid symptoms, either directly or indirectly, via specific cognitive or affective factors. Paranoid symptoms were measured using the SPEQ scale. Shorter sleep time and increased dreaming, mediated by both positive and negative affective states, predicted paranoid symptoms. Paranoid symptoms did not significantly predict sleep parameters.

### 4.2. Association of Insomnia with Suicidal Ideation and Suicide Attempts

Lack of sleep can increase the risk of suicide. Freeman and colleagues [68] explored the prevalence of suicidal ideation and behavior in 110 patients with persecutory delusions in the context of nonaffective psychosis (an umbrella diagnostic grouping that includes DD). The frequency of suicidal ideation and behavior, and its severity within the previous month, were evaluated by means of the Columbia Suicide Severity Rating Scale (C-SSRS). Psychopathological symptoms were assessed using the Beck Depression Inventory II (BDI-II), the Psychotic Symptom Rating Scales—Delusions (PSYRATS), and the Green Paranoid Thoughts Scale (GPTS), among other lesser known scales. Sleep difficulties correlated positively with the frequency of suicidal ideation.

González-Rodríguez and co-workers [69] reviewed the literature (10 studies) on the frequency of suicidal ideation and suicidal behavior in patients with DD. The frequency of suicidal behavior ranged from 8% to 21% and was more frequent in patients with persecutory and somatic delusions than in the other DD subtypes [67]. In a descriptive study of 44 cases, this research team also explored the association between comorbid depression and suicidal attempts in patients with DD [70]. The Positive and Negative Syndrome Scale (PANSS), the Personal and Social Performance Scale, and the Hamilton Depression Rating Scale (HDRS) were used to assess psychotic and depressive symptoms and social function. The Columbia Suicide Severity Rating Scale was used for the assessment of suicidal ideation and behavior. Patients with comorbid depression (CD) were found to score higher on the PANSS general subscale and also on the severity of suicidal ideation when compared to patients without CD. Although a direct association between insomnia and suicidal ideation and behavior was not investigated, this study showed that, in this population as in others, a high Hamilton score (which includes a measurement of insomnia) positively correlates with suicide risk.

### 4.3. Association of Insomnia with Cognitive Performance

Delusional disorder (DD) has been traditionally considered a psychotic syndrome that does not involve cognitive deterioration. However, when compared to healthy controls, individuals with DD do significantly less well on most cognitive tests when sex, age, educational level, and premorbid IQ are taken into account [71]. There is a strong relationship between memory processing and sleep characteristics in patients with psychosis, though it is more apparent in schizophrenia than in the broader psychosis spectrum, which encompasses DD [72].

In the general population, the relationship between sleep problems and cognitive deterioration has been demonstrated many times. A recent study of 99 healthy individuals 50 years of age or older investigated the association between sleep quality and visual and verbal memory [73]. The Pittsburgh Sleep Quality Index (PSQI) questionnaire was used to assess the quality of sleep and the Word Learning and Visual Paired Associates tests assessed visual and verbal memory. Scores on the cognitive tests decreased as sleep quality deteriorated. Sleep quality and memory impairment were investigated in a sample of 15,246 Chinese patients in the over 50 age group by Ma et al. [74]. In this study, cognitive performance was assessed by means of the Delayed Word Recall Test (DWRT) and the Mini Mental State Examination (MMSE), which both yield measures of memory impairment. Sleep quality was assessed by the PSQI questionnaire [72]. After controlling for potential confounding factors, sleep efficiency was found to be positively associated, in a dose–response pattern, with scores on memory impairment. On the other hand, Kang and colleagues [75] investigated the relationship between sleep disturbances and subjective memory complaints in 352 elderly Korean subjects and found that, while both depressive symptoms and subjective sleep quality were correlated with subjective memory complaints, no association with objective cognitive measures emerged from the data. The Korean version of the Consortium to Establish a Registry for Alzheimer’s Disease (CERAD-KN) was used to evaluate objective cognitive performance. Subjective memory complaints were assessed by the Subjective Memory Complaints Questionnaire (SMCQ), and sleep quality by means of the Pittsburgh sleep quality index. Subjective memory deficits, while not objectively measurable, are nevertheless considered potential early signs of impending cognitive deterioration.

In 2020, Kumari and Ettinger [76] reviewed all sleep deprivation studies in healthy volunteers and concluded that sleep loss was associated not only with psychotic symptoms such as paranoid and suicidal ideation and cognitive deterioration in executive function, attention, memory, language, and social responsiveness, but also with electrophysiological biomarkers for psychosis such as prepulse inhibition, antisaccades, and smooth pursuit eye movements. Furthermore, the longer the sleep deprivation, the more detrimental its effect. The speculation is that disrupted dopamine pathways serve as the link between sleep and psychotic manifestations [42].

## 5. Association of Psychosis Treatment with Sleep Disturbance

Antipsychotic medications interact with many neurotransmitter systems, with a main effect on psychotic symptoms mediated via blockade of the dopamine D2 receptor on postsynaptic neurons. The therapeutic use of these drugs in schizophrenia is closely associated with the presence of hypersomnia, sleep paralysis, sleep apnea, bruxism, and restless leg syndrome. Some APs, such as aripiprazole and ziprasidone, have been associated with insomnia [77]. Patients with DD receive the same antipsychotic treatment as patients with schizophrenia, with doses usually being similar [78]. Due to the fact that sleep and wakefulness are partly controlled by dopamine [79], a putative link exists between sleep disturbance and AP treatment, although sleep problems in psychiatric illness frequently occur in the absence of any drug treatment [42]. Table 3 summarizes some of the sleep conditions that have reportedly been aggravated by antipsychotics.

## 6. Therapeutic Options for Delusional Disorder via Improvements in Sleep

Due to the fact that sleep disturbances increase the severity of psychotic symptoms, it is possible that the effective treatment of sleep problems can also serve as an adjunctive treatment for psychosis [85,86]. Accurate diagnosis of the precise sleep difficulty is needed first. This can be achieved via inspection of polysomnography parameters, spectral analytic data, and subjective sleep estimates [87]. Due to the fact that women show deeper sleep compared to men and sleep patterns change as one grows older [87], the selection of a therapeutic intervention also depends, among other factors, on variables such as sex and age.

In an effort to modulate sleep spindles and wave oscillations during sleep in schizophrenia, a variety of neurostimulation techniques have been proposed (electrical, magnetic, and radio frequency stimulations of auditory, olfactory, visual, and vestibular pathways) [28,88,89]. The hypothesis is that correcting deficits in sleep parameters will improve cognition impairments. This promising area is, however, still in its infancy. With respect to pharmacotherapy, the British Association for Pharmacology has issued recent guidance on the use of sleep medications for patients with psychosis, for those with circadian rhythm disturbance, and for those with parasomnias [90]. It is not, however, clear what pharmaceuticals are most useful for patients who fall into all three categories. Treatments targeting insomnia and nightmares that are currently supported by the most evidence are psychological e.g., cognitive behavioral therapies (CBT) [85,86,91]. Response is, however, variable [92,93].

Sheaves and collaborators [94] carried out an assessor-blind pilot two-week randomized controlled trial in 40 patients admitted to hospital in acute psychiatric crisis. Participants were randomized to receive CBT plus standard care (SC) versus SC alone. Exposure to light and dark was combined with the CBT to ensure circadian entrainment. Primary outcome measures were the Insomnia Severity Index and Warwick–Edinburgh Mental Wellbeing Scale. Patients receiving CBT plus light/dark exposure showed greater reductions in insomnia than those treated with SC, though improvement in mental wellbeing was very slight. The participants of this study were suffering an acute psychiatric crisis, precise diagnosis unknown.

Freeman et al. [95], in a single-blind randomized trial of UK university students, showed that treatment of insomnia diminished paranoia and hallucinations. In 2015, the same research group conducted a prospective, assessor-blind, randomized controlled trial, the Better Sleep Trial (BEST), that included a 24-week follow-up assessment [96]. In that trial, the participants were patients with insomnia and persistent delusions who were randomized to CBT or standard care. Degree of insomnia was assessed by the Insomnia Severity Index and psychotic symptoms were assessed by the Psychotic Symptoms Rating Scale. The findings were that CBT significantly improved sleep and diminished the frequency of paranoid and hallucinatory experiences in patients with persistent delusions. The participants in that study were all diagnosed with schizophrenia, so the findings may or may not be applicable to DD.

Sheaves and collaborators [91] carried out an assessor-blind parallel-group pilot randomized trial of 24 patients with persistent persecutory delusions and nightmare disorder. Participants were randomly assigned to CBT for nightmares plus treatment as usual (TAU) or TAU alone for four weeks. Assessments took place at baseline, at four weeks (end of treatment), and again eight weeks later. The following psychometric instruments were used: The Disturbing Dream and Nightmare Severity Index (DDNSI) for evaluating nightmare severity, the Sleep Condition Indicator (SCI) for Insomnia, the Pittsburg Sleep Quality Index (PSQI) for sleep quality, the Depression, Anxiety, and Stress Scale (DASS-21) for affective symptoms, the Green Paranoid Thoughts Scale (GPTS) for paranoid symptoms, the Cardiff Anomalous Perceptions Scale (CAPS) for anomalous senso-perceptive experiences, and the Beck Suicide Scale (BSS) for suicidal ideation. At the end of therapy, patients on CBT showed more improvement in nightmare frequency than those treated with TAU, and they also showed steeper reductions in paranoid symptoms. The clinical improvement was maintained at eight-week follow-up (and, as a bonus, was also associated with substantial improvement in insomnia).

There are many therapies for insomnia, nightmare disorder, sleepwalking, sleep apnea, bruxism, sleep paralysis, restless leg syndrome, and hypersomnia [7,97,98] that have not yet been trialed in patients with delusions. The possibility remains open that some of these treatments could, by increasing the quality of sleep, reduce psychotic phenomena.

## 7. Discussion

What we learned from this literature review is that individuals with persistent delusions commonly suffer from sleep disturbances and that regulating sleep may reduce the severity of psychotic symptoms and perhaps also help to correct cognitive deficiencies and decrease suicidal urges. The scientific literature supports a convincing association between sleep disturbances, cognitive impairment, and psychopathological symptoms (particularly, paranoid symptoms and suicidal ideation) in patients with delusions. What needs to be further researched is the gap between subjective sleep quality and objective sleep measures [99]. A recent study investigated the relationship between electroencephalogram (EEG) power spectral density and subjective sleep quality in healthy individuals [100]. Effect sizes of the correlations between the two were small, suggesting that subjective and objective sleep may be somewhat different constructions.

There are many pitfalls to sleep treatment in this population because a delusional person can misinterpret a clinician’s intent and refuse to co-operate with therapy. The first step to successful treatment is to establish a therapeutic relationship of trust [101]. The content of the delusional belief needs to be considered. A suspicious person may, for fear of nocturnal attack, prefer a light sleep, from which he or she can be easily awakened. A person tormented with jealousy may also feel the need to be constantly vigilant, perhaps particularly at night [102]. Psychological obstacles such as these require thorough knowledge of individual circumstances before any treatment is proposed. While effective treatment of sleep disturbance promises to diminish delusional and related symptoms, there remain many clinical challenges. An early challenge is gaining the patient’s trust so that therapeutic strategies can be initiated. A second challenge is the necessary but potentially aggravating role played by psychotropic medication. The risks and benefits of all prescribed medications need to be carefully evaluated. A third clinical challenge is to refrain from attributing all symptoms presented by persons with psychotic disorders to their mental disorder. All comorbidities that can benefit from targeted treatment need to be addressed. Prolonged changes in auxiliary symptoms, not only in sleep patterns but also in appetite, weight, and physical activity may instigate/exacerbate psychotic phenomena. The hope is that optimal management of such “secondary” symptoms will ameliorate personal and interpersonal functioning and have a spillover beneficial effect on primary symptoms. Close liaison between mental health and sleep specialists is fundamental to the achievement of good results.

## 8. Conclusions

Sleep is important to mental health. There is accumulating evidence that sleep disturbances contribute to the occurrence of mental health problems and to increased severity of many psychopathological symptoms. While there are persuasive reports of sleep disorders and their treatment positively impacting the psychopathology and clinical course of patients with schizophrenia and affective disorders, this connection has not been adequately studied in other psychotic disorders. The precise prevalence of sleep disturbances in delusional disorder is unknown, as is the strength of the link between sleep disorders and paranoid symptoms, suicidal ideation, and cognitive performance. Preliminary evidence does, however, suggest a bidirectional link, with a potential for crossover therapies.

## Figures and Tables

**Table 1:** Diagnostic criteria for delusional disorder (DD).

Diagnostic Criteria for DD
One (sometimes more) delusion(s) persisting for at least one month
Full criteria for schizophrenia have never been met
Functioning is not obviously impaired and behavior is not noticeably aberrant
Affective episodes, if any, are brief
The disturbance cannot be attributed to any other physical or mental disorder
The delusion can be moderate or severe, bizarre or non-bizarre

**Table 2:** Subtypes of DD.

Subtypes of DD
Erotomanic type: Harboring a false conviction of someone having fallen in love with them
Grandiose type: Falsely believing that they are persons of special importance
Jealous type: Harboring a false conviction that their lover is unfaithful
Persecutory type: Falsely believing that they are being conspired against, spied on, poisoned, harassed or followed
Somatic type: Harboring false convictions about having a medical condition, misinterpreting perceptions or body sensations
Mixed type: Delusions that incorporate elements of several of the above
Unspecified type: The dominant delusional belief cannot be determined or falls outside of those specified above

**Table 3:** Sleep conditions exacerbated by antipsychotics.

Sleep Disturbance	Definition
Hypersomnia	Excessive sleepiness aggravated by antipsychotic-induced sedation [44]
Restless Leg Syndrome	An urge to move the legs at night aggravated by antipsychotic-induced akathisia [80]
Bruxism	Tooth grinding or clenching during sleep, aggravated by antipsychotic-induced dyskinesia [81]
Sleep Apnea	Pauses in breathing at night, aggravated by antipsychotic-induced weight gain [82,83,84].
